# Differentiating BPD in adolescents with NSSI disorder: the role of adverse childhood experiences and current social relationships

**DOI:** 10.1186/s40479-018-0097-5

**Published:** 2018-12-07

**Authors:** Christel J. Hessels, Odilia M. Laceulle, Marcel A. G. van Aken, Franz Resch, Michael Kaess

**Affiliations:** 1 Psychiatric Center GGz Centraal, Amersfoort, Netherlands; 2 Department of Developmental Psychology, Utrecht University, Utrecht, Netherlands; 3 Clinic of Child and Adolescent Psychiatry, Center for Psychosocial Medicine, University Hospital Heidelberg, Heidelberg, Germany; 4 University Hospital of Child and Adolescent Psychiatry and Psychotherapy, University of Bern, Bern, Switzerland; 5 Section for Translational Psychobiology in Child and Adolescent Psychiatry, Department of Child and Adolescent Psychiatry, Center for Psychosocial Medicine, University of Heidelberg, Heidelberg, Germany

**Keywords:** Borderline personality disorder (BPD), Nonsuicidal self-injury (NSSI), Adverse childhood experiences, Relationship quality, Adolescent

## Abstract

**Background:**

As borderline personality disorder (BPD) is increasingly considered a lifespan developmental disorder, we need to focus on risk factors and precursors in the developmental pathways to BPD, in order to enable early detection and intervention. Within this developmental pathway, adolescence is a crucial phase in the light of the manifestation of the disorder. Relational factors such as adverse childhood experiences and current relational problems can be considered important in adolescents who are at-risk for BPD. Nonsuicidal self-injury (NSSI) is a key precursor for adolescent BPD and one of the most promising targets for early detection and intervention of BPD.

**Methods:**

In a clinical sample of 152 adolescents engaging in nonsuicidal self-injury (NSSI) disorder referred to mental healthcare in Germany, this study investigated whether we can differentiate who has BPD from 1) adverse childhood experiences; and 2) the quality of current relationships, both with parents and peers. BPD was assessed both categorically as a dichotomized score and dimensionally as a continuous score.

**Results:**

More adverse childhood experiences, but not low quality of current social relationships, were related to more BPD symptoms and an increased risk for meeting full criteria for BPD. In the dimensional model, current social relationship quality with parents and peers did not show a moderating (protecting or aggravating) effect on the association between adverse childhood experiences and BPD. Using a categorical approach, however, the association between childhood adversity and meeting full criteria for BPD was higher in individuals reporting higher quality of current parent-child relationship.

**Conclusions:**

These results highlight adverse childhood experiences as risk factors of BPD, while the role of current social relationships seems more complex.

## Background

### Adolescent borderline personality disorder and nonsuicidal self-injury

Over the last decades, reluctance and ambivalence in assessing and diagnosing borderline personality disorder (BPD) in young people has shifted to a view in which personality disorders are being considered lifespan developmental disorders with possible precursors early in life. This shift has several implications: There is an increasing integration of developmental research which traditionally focused more on personality dimensions [1]. A dimensional perspective [2], may better account for the developmental fluctuations and increased heterogeneity that have been reported especially in younger samples [3]. Thus, the way is paved to consider personality disorders from a developmental psychopathology perspective. Although we still have limited data available on the developmental mechanisms specifically associated with BPD [4], current literature focuses on the identification of risk factors and precursors that play a role in the developmental pathways or mechanisms leading to BPD, specifically in younger populations, such as dysregulated behavior in childhood, family adversity, maladaptive mother-child interactions [6], bullying behaviour [5, 7] and childhood nightmares [8]. Finally, the developmental pathways can be understood by examining the dynamic interaction of normal and abnormal biological, psychological, and sociocultural factors and systems over critical developmental periods across the life course [5]. This dynamic interaction can be understood as for example children demonstrating higher levels of childhood dysregulation are prone to the development of BPD symptoms when exposed to environmental risk factors, but also dysregulated children seem to be more likely to be exposed to these environmental risks [9]. This means that within this pathway individual factors and psychosocial factors influence each other reciprocally. For example, although both poor self-control and harsh parenting in 5–14 years old girls predicted the development of BPD symptoms at age from 14 to 17, both factors were partially mediated by their earlier reciprocal effects on each other between ages 5 and 14 [10]. Furthermore, an indirect association between childhood dysregulation and BPD via an increased risk of bullying was found [7].

Self-harm is highly associated with BPD, both in adults [8] and in adolescents [9], and is defined in terms of both suicidal behavior and nonsuicidal self-injury (NSSI). While rates of self-harm tend to decline in individuals with BPD towards adulthood [10, 11], the BPD criterion ‘self-harm and suicidal behavior’ is the one that is most frequently met in adolescents with BPD [12]. In addition, to being a common symptom of BPD, NSSI is often present prior to being diagnosed with full-blown BPD. Specifically, as up to 30% of adults with BPD reported the onset of self-harm prior to the age of 12, and another 30% between the ages 13–17, self-harm can be considered to be a key precursor for BPD [5, 13]. Self-harm in general, and NSSI in particular [13], are serious health problems [14]. However, roughly 50% of adolescent and adult patients with NSSI do not meet the diagnostic criteria for BPD (e.g., [15]). This has led to a discussion whether NSSI should be considered as a distinct and clinically significant diagnostic entity [15], and to the inclusion of the newly diagnostic entity of non-suicidal self-injury (NSSI) disorder in Section III of DSM-5 [2]. Especially in adolescence, the relation between BPD and NSSI is complex. Although NSSI is common among adolescents and young adults and is associated with a range of clinical syndromes, there is evidence that particularly repetitive and long-lasting NSSI might be a precursor for BPD [16].

Overall, adolescents with NSSI can be considered an important group at-risk for developing or already suffering from BPD but only a proportion of adolescents with NSSI have or will develop BPD. Therefore, understanding associations between different developmental and psychosocial factors within a critical phase of the developmental pathway to BPD is highly relevant, specifically in a high-risk population such as adolescents with NSSI-disorder.

### Adverse childhood experiences in relation to BPD and NSSI-disorder

BPD and NSSI-disorder can be seen as developing against the background of profoundly disturbed interpersonal experiences [4]. Adverse childhood experiences mainly take place within an interpersonal context and are considered a risk factor for BPD as well as for NSSI and suicidal behavior [17, 18]. There is substantial evidence that adverse childhood experiences, in particular emotional neglect and sexual abuse, are associated with BPD [18–20]. For example, the Children in the Community Study found that documented childhood maltreatment was prospectively associated with a highly increased risk for BPD in young adulthood, even when controlling for symptoms of other personality disorders, age, parental education and parental psychiatric disorders [21]. Lyons-Ruth et al. [22] suggested that to best account for borderline symptoms, models need to include both abuse experiences and aspects of early parent-infant interactions and that repeated parent-child assessments are needed to fully account for the emergence of BPD.

The precise role of adverse childhood experiences in the etiology of BPD is not clear, because putative risk factors, such as childhood maltreatment, parental bonding difficulties, and adverse familial environment, might all contribute to the development of BPD and are often highly intercorrelated. Infurna et al. [18] found that, although highly correlated among each other, sexual abuse, low care from the mother and negative general functioning all independently contributed to BPD development. Less attention is paid in the literature to the interaction of highly correlated adverse childhood experiences with current social relationships. Especially in adolescents, it seems important to not only study specific childhood adversities, but also study these adversities in the context of adolescents’ current family and social relations.

Within the aetiology of both NSSI and BPD, adverse childhood experiences, such as parental antipathy or neglect as well as sexual abuse are found to be risk factors [7, 18, 19] as well as bullying in childhood [7], specifically considering the role of adverse childhood experiences in NSSI, research has shown that both parent and peers support was found to be protective for self-harm after being bullied [10].

### The role of social relationships

Both inside and outside the family, social interactions and social support are important for the development of personality in young people [23]. Problems in social functioning and social relations are considered key elements for understanding the course of personality disorders [24]. Moreover, Chanen and Kaess [5] stated that in contrast to the relatively unstable nature of the diagnosis BPD, both in adolescents and in adults, problems in social functioning seem to be relatively stable and may have long-lasting consequences for the individual’s functioning.

Findings about whether the social environment, plays a role in the development of subsequent problems for maltreated children are heterogeneous and contradictory [18] and as far as we know have focused less on emerging BPD. Perceived social support is conceptualized as a mediating variable in the relation between childhood physical abuse, sexual abuse, and neglect and developmental achievement [25], posttraumatic stress disorder [26] and depression [27]. In addition, social relationships seem so be a protective factor for NSSI. In a recent review, Mummé, Mildred and Knight [28] found that interpersonal factors, such as family support and social connectedness and intrapersonal factors, such as self-esteem and emotional regulation, facilitated the cessation of NSSI, with family support being the predominant interpersonal factor in influencing NSSI cessation.

Understanding particularly early relational experiences is important to be able to reduce environmental risks early in the course of the developmental pathway of BPD, where NSSI disorder can be considered as a precursor [5]. In addition, the quality of current social relationships is an important factor to consider in adolescence [5]. The expanding research on this topic is necessary to inform the development of prevention, early detection, and timely intervention for BPD [6, 7]. As far as we know, the relation between adverse child experiences and BPD within a high risk population for its development, as well as the buffering effect of social support has not been studied yet.

### Current study

The current study aims at increasing our understanding of adverse childhood experiences, current relational functioning and BPD in a sample of adolescents with NSSI. Within the overall group of adolescents with NSSI, it is important to be able to distinguish those who are at risk for developing BPD, so we will be able to think of appropriate intervention for early intervention or even indicated prevention. Specifically, within a clinical sample of 166 adolescents with NSSI-disorder referred to mental healthcare in Germany, the following research questions will be answered:

1. How are adverse childhood experiences related to BPD in this at-risk group?
2. How is the quality of current relationships with parents and peers related to BPD in this at-risk group?
3. Is the link between adverse childhood experiences and BPD moderated by the quality of current relationships to both parents and peers?

It is hypothesized that more adverse childhood experiences and/or lower quality of current relationships are related to more BPD in adolescents with NSSI. Whether the quality of current relationships moderates the link between adverse childhood experiences and BPD will be explored. However, based on previous literature on the buffering effects of social relations it is expected that individuals who report more adverse experiences have less BPD if they report a higher quality of current relationships. Additionally, given the recent shift in the BPD literature from a DSM oriented, categorical approach to a more dimensional, continuous approach, special attention will be paid to the possible additive value of the dimensional, to the traditional categorical approach.

## Methods

### Participants

This study is part of an ongoing clinical cohort study within AtR!Sk (*“Ambulanz für Risikoverhalten und Selbstschädigung”)*, an outpatient program for early identification and intervention of BPD at the Department of Child and Adolescent Psychiatry of the University Hospital Heidelberg. The measures for the study were part of the structured clinical assessment at entry to AtR!Sk. Participants seeking help for any risk-taking and self-harm behavior within AtR!Sk were recruited consecutively into the AtR!Sk cohort study. Participating in the research meant giving informed consent from both patients and caregivers that the data could be used anonymously for research purposes. The study was approved by the respective Ethics Committee of the Faculty of Medicine. Risk-taking and self-harm was defined as; NSSI; suicidal behavior, binge drinking, substance misuse, excessive internet or media use, sexual risk-behavior, as well as impulsive high-risk and delinquent behavior. The only exclusion criterion was lack of language comprehension. Within a recruitment period of 27 months, 246 adolescents entered the diagnostic stage of AtR!Sk. Out of those, a total of 221 individuals (89.8%) participated in the ATR!Sk cohort study. The mean age of participants was 15 years (*M* = 15.07; *SD* = 1.4, range 11–17), and they were mostly girls (184 girls, 83.3%; 37 boys, 16.7%). For the current study, we included all participants who had injured themselves without suicidal intent on 5 or more days in the last 12 months and therefore, met the criteria of NSSI disorder. This resulted in a sample of 166. However, because of missing data, the sample for the different research variables was152 (see statistical analyses).

### Measures

#### Nonsuicidal self-injury

NSSI was operationalized with the German translation of Self-Injurious Thoughts and Behaviors Interview (SITBI; [29]), a structured interview which assesses the presence, frequency, severity, age-of-onset, and other characteristics of NSSI, suicidal ideation, suicide plans, suicide gestures, and suicide attempts. Fischer et al. [30] found good psychometric properties of the SITBI-G, which were comparable to the original SITBI interview. The interrater reliability was very good (average κs = .77–1.00). Construct validity ranged from moderate to good agreements. For this study, the SITBI was modified in accordance with the DSM-5 criteria. We used the total number of days of engagement in NSSI in the past year. Participants who had injured themselves on 5 or more days met the criteria of NSSI-disorder according to DSM-5 and were included in the further analysis (*N* = 166).

#### Borderline Personality Disorder

BPD was operationalized according to the BPD scale of the German translation of the SCID-II interview [31]. Interview items are coded using codes of 1 = absent or false (a criterion symptom for disorder clearly absent), 2 = subthreshold (criterion threshold almost, but not quite met), 3 = threshold or true (criterion threshold is met). In the analyses both a dichotomized score for full BPD was used, which reflects 5 or more criteria which met criterion threshold (score 3) and a dimensional scale, which reflected the number of criteria which met the threshold. Finally, in the multinomial regression we used 3 groups; no BPD (0–2 criteria above threshold); subthreshold BPD (3–4 criteria above threshold) and full BPD (≥5 criteria above threshold).

#### Adverse Childhood Experiences

Adverse Childhood Experiences were reported retrospectively with a German translation of the Childhood Experiences of Care and Abuse Questionnaire (CECA.Q), which measures adverse childhood experiences in the period prior to age 17 [32, 33]. Physical and sexual abuse are assessed with screening questions, while antipathy and neglect are measured by scales repeated for mother and father independently. We aggregated the scores on parental loss due to death of a parent and separation over a year under the age of 17 years to the factor parental loss. The scores on antipathy, neglect, parental loss, physical abuse by a parent, and sexual abuse were aggregated to one dimension ‘Adverse Childhood Experiences’, by computing the mean score of the dichotomized variables, when at least 3 items had scores. The German translation of the CECA.Q showed good internal consistency (Cronbach’s alpha from 0.86 to 0.93) and adequate test-retest reliability (Cohen’s *k* from 0.78 to 0.93) [33].

#### Quality of current relationships with parents and peers

Quality of current relationships with parents and peers were measured with two dimensions from the German translation of the KIDSCREEN-52; Gesundheitsfragebogen für Kinder und Jugendliche asking the participants perceptions of the last week [34]: Parent relation and home life (examples of questions were: ‘Have your parents had enough time for you?’; ‘Have you been able to talk to your parents when you wanted to?’; Cronbach’s alpha: 0.90); and Social Support and Peers (examples of questions were: ‘Have you had fun with your friends?’; ‘Have you been able to rely on your friends?; Cronbach’s alpha: 0.86). A European survey involving 12 countries (i.e., Austria, Switzerland, Czech Republic, Germany, Greece, Spain, France, Hungary, The Netherlands, Poland, Sweden and the UK) and 22,110 children and adolescents aged between 8 and 18 years of age, showed that this questionnaire is a good cross-cultural measure of health-related quality-of-life assessment for children and adolescents in Europe [35].

### Statistical analyses

First, several descriptive statistics were calculated for the full sample of patients with NSSI disorder, the subgroup who met the full criteria for BPD and the subgroup who did not meet the full criteria for BPD. Bivariate correlations (using pairwise deletion) were calculated between gender, age, BPD (both categorical, differentiating syndromal BPD (≥5 DSM-5 BPD criteria) from subsyndromal (< 5 DSM-5 BPD criteria) BPD, and continuous), adverse childhood experiences, parent relationships and social support.

Second, logistic regression was used to predict the dichotomized score for BPD (1 = full BPD, *N* = 82; 0 = subsyndromal BPD, *N* = 70) by adverse childhood experiences and quality of current relationships in individuals with NSSI disorder (*N* = 152). In this analysis, BPD was regressed in separate blocks. In block 1, adverse childhood experiences were added, in block 2 quality of current relationships with parents and peers, and in block 3 the interaction terms of adverse childhood experiences and respectively quality of current relationships with parents, and with peers. To prevent effects of multicollinearity, one interaction term was added at a time (i.e., first: adverse experiences X parent relationships, second: adverse experiences X peer relationships). Gender and age were taken into account as confounding variables in all analyses prior to adding any of the other variables (block 0).

Third, hierarchical regression analyses were used to examine the link with the same variables as the logistical regression models, but this time regressing the continuous score of BPD criteria. That is, this continuous score was again regressed on gender, age, mean adverse childhood experiences, quality of parent relationships, quality of peer relationships and the interaction terms (one at a time).

Fourth, post hoc multinomial regression analyses were used to examine whether the same variables as in the previous regression analyses could predict variability in the full BPD group (≥5 DSM-5 criteria) versus the group of no BPD (1–2 DSM-5 criteria) or the subthreshold group (3–4 DSM-5 criteria).

## Results

### Descriptive statistics

Descriptive statistics of age, gender differences and the various research variables as well as the type, severity and frequency of self harm for the group with BPD and the group who did not meet the criteria for full threshold BPD are shown in Table 1. Correlation coefficients for the full sample are reported in Table 2. Most important for our research questions, adverse childhood experiences were related to more BPD symptoms (both continuous (*r* = .30, *p* < .05) and categorical (*r* = .27, *p* < .05)), whereas quality of current parent and peer relationships were not related to either operationalization of BPD.

**Table 1:** Descriptives of research variables for the total NSSI disorder sample, BPD and no-BPD Group, respectively

Research variable	Total sample NSSI disorder (*N* = 166)		BPD (*N* = 93)		No BPD (*N* = 73)	
	*N*	%	*N*	%	*N*	%
Gender (*N*, % = Female)	151	91.0	90	96.8	61	83.6
	M	SD	M	SD	M	SD
Age	15.04	1.34	15.29	1.29	14.73	1.35
Number of BPD criteria	4.72	2.00	6.16	1.22	2.89	1.10
BPD diagnostic criteria	N	%	N	%	N	%
Fear of abandonment	57	34.3	47	50.5	10	13.7
Unstable relationships	96	57.8	77	82.8	19	26.0
Identity disturbances	64	38.6	55	59.1	9	12.3
Impulsivity	42	25.3	35	37.6	7	9.6
Self-harm/Suicidality	164	98.8	93	100.0	71	97.3
Affective instability	118	71.1	88	94.6	30	41.1
Inner emptiness	103	62.0	70	75.3	33	45.2
Inappropriate anger	74	44.6	58	62.4	16	21.9
Paranoia/Dissociation	66	39.8	50	53.8	16	21.9
Adverse Childhood Experiences	N	%	N	%	N	%
Antipathy Mother	64	38.6	43	46.2	21	28.8
Antipathy Father	68	41.0	44	47.3	24	32.9
Neglect Mother	36	21.7	20	21.5	16	21.9
Neglect Father	56	33.7	37	39.8	19	26.0
Parental Loss	54	32.5	33	35.5	21	28.8
Physical Abuse	42	25.3	29	31.2	13	17.8
Sexual Abuse	43	25.9	33	35.5	10	13.7
Current social functioning	M	SD	M	SD	M	SD
Parent relationship Quality	2.91	1.04	2.82	1.01	3.02	1.08
Peer Relationship Quality	3.10	.94	3.15	.92	3.04	.96
Type of non suicidal self harm	N	%	N	%	N	%
Cut or carve skin	165	99.4	92	98.9	73	100.0
Skin scraping	91	54.8	52	55.9	39	53.4
Wound picking	82	49.4	47	50.5	35	47.9
Skin burning	65	39.2	43	46.2	22	30.1
Deliberate self-hitting	54	32.5	33	35.5	21	28.8
Biting	51	30.7	30	32.3	21	28.8
Severity of Self harm	N	%	N	%	N	%
In need for treatment after self-harm	44	26.5	31	33.3	13	17.8
Frequency of Self harm	M	SD	M	SD	M	SD
Thoughts on self-harm last month	22.35	45.66	26.43	58.97	17.11	16.55
Self-harming behaviours last month	9.22	12.71	9.80	14.79	8.49	9.46
Reported reason for self-harm	N	%	N	%	N	%
Mental State	138	83.1	77	82.8	61	83.6
Dispute with parents or family	114	68.7	47	50.5	48	65.8
School distress	78	47.0	42	45.2	36	49.3
Dispute with friends	74	44.6	47	50.5	27	37.0
Dispute with best friend	42	25.3	28	30.1	14	19.2
Bullied/eviction	46	27.7	26	28.0	20	27.4

**Table 2:** Pearson Correlations between Borderline Personality Disorder and Predictor Variables (*N* = 166)

	1	2	3	4	5	6
1 Gender	–					
2 Age	.05	–				
3 BPD (dimension)	**−.23**	**.29**	–			
4 BPD (diagnosis ≥5 criteria)	**−.23**	**.21**	**.81**	–		
5 Childhood Adverse Experiences	−.09	.06	**.30**	**.27**	–	
6 Parental Relationship Quality	.11	−.02	−.15	−.10	**−.68**	–
7 Peer Relationship Quality	.01	*.18*	−.06	.06	*−.18*	*.17*

Note: **bold** values are significant *p* < .01, *italic* values are significant *p* < .05. N ranges between 148 and 166

### Main analyses

#### Categorical approach

A logistic regression was conducted to predict the dichotomized score of BPD (1 = full BPD, 0 = subsyndromal) by adverse childhood experiences and quality of parent and peer relationship as well as the interaction variables as predictors. Model statistics and path estimates are reported in Table 3.

**Table 3:** Summary of Logistic Regression Analysis for (the cumulative effect of) Adverse Childhood Experiences and Parent as well as Peer Relationship Quality Predicting Categorical Borderline Personality Disorder (BPD > 4 criteria) and Hierarchical Regression Coefficients of the Relationship Between the predicting variables, and Dimensional Borderline Personality Disorder (N = 152)

		Categorical BPD					Dimensional BPD					Collinearity Stat.	
		*B*	*SE B*	*EXP (B)*	*p*	95% CI *B*	*B*	*SE B*	*EXP (B)*	*p*	95% CI *B*	Tolerance	VIF
Step 1	Age	.41	.13	1.50	**.002**	1.16, 1.94	.47	.11	.31	**.000**	0.25, 0.70	0.998	1.002
	Gender	−1.87	.69	.15	**.006**	0.04, 0.59	−1.66	.52	−.24	**.002**	−2.69, −0.64	0.998	1.002
Step 2	Age	.41	.14	1.50	**.003**	1.15, 1.96	.45	.11	.30	**.000**	0.23, 0.67	0.993	1.007
	Gender	−1.84	.71	.16	**.009**	0.04, 0.64	−1.50	.50	−.22	**.003**	−2.50, −0.51	0.989	1.011
	Adverse Childhood Experiences	.89	.31	2.42	**.004**	1.33, 4.41	.85	.24	.26	**.001**	0.37, 1.33	0.988	1.012
Step 3	Age	.39	.14	1.48	**.005**	1.12, 1.95	.47	.11	.31	**.000**	0.24, 0.69	0.955	1.047
	Gender	−1.93	.73	.15	**.008**	0.04, 0.60	−1.55	.50	−.23	**.003**	−2.54, −0.55	0.985	1.015
	Adverse Childhood Experiences	1.37	.44	3.95	**.002**	1.67, 9.33	1.03	.33	.31	**.002**	0.38, 1.69	0.531	1.882
	Parent Relationship Quality	.37	.24	1.45	.124	0.90, 2.34	.21	.20	.11	.293	−0.18, 0.59	0.539	1.856
	Peer Relationship Quality	.13	.21	1.14	.540	0.76, 1.70	−.16	.17	−.07	.354	−0.49, 0.17	0.923	1.084
Step 4a	Age	.39	.14	1.48	**.005**	1.16, 2.07	.48	.11	.32	**.000**	0.26, 0.71	0.942	1.062
	Gender	−1.93	.73	.15	**.008**	0.03, 0.52	−1.61	.51	−.24	**.002**	−2.61, −0.61	0.977	1.024
	Adverse Childhood Experiences	1.37	.44	3.95	**.002**	1.86, 10–75	1.10	.33	.33	**.001**	0.44, 1.76	0.521	1.920
	Parent Relationship Quality	.37	.25	1.45	.130	0.97, 2.63	.25	.20	.13	.208	−0.14, 0.64	0.526	1.900
	Peer Relationship Quality	.13	.21	1.13	.541	0.78, 1.79	−.14	.17	−.06	.410	−0.46, 0.19	0.918	1.090
	ACE X Parent Relationship Quality	.59	.30	1.81	**.048**	1.01, 3.25	.34	.24	.11	.157	−0.13, 0.82	0.945	1.058
Step 4b	ACE X Peer Relationship Quality	−.01	.35	.99	.971	0.50, 1.96	−.13	.27	−.04	.639	−0.66, 0.51	0.971	1.030

Note: **bold** values are significant *p* < .05. Nagelkerkes R^2^ = .15 for Step 1; Nagelkerkes R^2^ = .22 for Step 2; Nagelkerkes R^2^ = .24 for Step 3; Nagelkerkes R^2^ = .24 for Step 4; R^2^ = .150 for Step 1; R^2^ = .214 for Step 2; R^2^ = .224 for Step 3; R^2^ = .235 for Step 4

Gender and age were taken into account in the first step of the analyses. Findings showed that males were less likely to have BPD than females (EXP(B) = .15), and older individuals more likely than younger individuals (EXP(B) = 1.50). Results also showed that adolescents who reported more adverse childhood experiences had an increased chance of meeting full criteria for BPD, compared to adolescents who experienced less childhood adversity (EXP(B) = 2.42). Specifically, with each standard deviation increase in the number of adverse childhood experiences, an individual is 2.42 times more likely to develop full criteria for BPD. Together, this model explained 22% of the explained variance (Nagelkerkes *R*^*2*^ = .22). Parent and peer relationship quality was not related to the likelihood of having BPD, and as such, did not significantly contribute to the model (Nagelkerkes *R*^*2*^ = .24). However, the link between adverse childhood experiences and BPD was moderated by parent relationship quality (but not peer relationship quality). That is, adverse childhood experiences showed a slightly stronger association with BPD in the presence of *good* relationships with parents (EXP(B) = 1.81; Nagelkerkes *R*^*2*^ = .24). This suggests that good parent relations *aggravate* (in contrast to buffer) the effect of adverse childhood experiences on the likelihood of meeting full criteria for BPD. The strong inverse correlation (*r* = −.68) between childhood adversity and parent relationship quality, however suggests that individuals who experienced more childhood adversity also report low quality of current relations with parents.

#### Dimensional approach

The hierarchical regression analysis predicting the number of BPD symptoms largely confirmed the findings of the logistic regression analysis. Results showed significant contributions of the confounders gender and age (*F* (2, 149) = 13.10, *p* < .001; *R*^*2*^ = .150) and adverse childhood experiences: (*F* (3, 148) = 13.47, *p* < .001; *R*^*2*^ = .214), but not of parent and/or peer relationship quality (*F* (3, 148) = 13.47, *p* < .001; *R*^*2*^ = .214). Thus, individuals who reported more adverse childhood experiences had more BPD symptoms. Adding the interaction terms to the model did not result in significantly more explained variance. Path estimates are reported in Table 3.

#### Full BPD, Subthreshold BPD and No BPD

The lack of interaction with parent relationship quality seems to contradict the logistic regression analysis demonstrating a moderating effect of parent relations. To clarify this difference, a multinominal regression analysis was conducted to investigate whether the main differences could be explained by the variability in the full BPD group (≥5 DSM-5 criteria) versus the subthreshold group (3–4 DSM-5 criteria) or the no BPD group (1–2 DSM-5 criteria). Results, reported in Table 4 showed significant effects of age (*χ*^*2*^ (2) = 11.89, *p* = .003), sex (*χ*^*2*^ (2) = 12.72, *p* = .002), adverse childhood experiences (*χ*^*2*^ (2) = 13.34, *p* = .001) and quality of peer relations (*χ*^*2*^ (2) = 10.86, *p* = .004). No significant effect of quality of parental relations (*χ*^*2*^ (2) = 3.73, *p* = .155) was found. Also, no effects of the interactions between adverse childhood experiences and quality of parent relations (*χ*^*2*^ (2) = 4.82, *p* = .090) or between adverse childhood experiences and quality of peer relations (*χ*^*2*^ (2) = .54, *p* = .762) were found.

**Table 4:** Summary of Multinominal Regression comparing Adverse Childhood Experiences and Parental as well as Peer Relationship Quality between no BPD (0–2 criteria; *N* = 22), subthreshold BPD (3–4 criteria, *N* = 48 with full BPD (≥5 criteria; *N* = 82)

	95% Cl for Odds Ratio					
	B	*SE B*	Lower	Odds Ratio	Upper	*p*
No versus Full BPD						
Sex	−2.95	0.91	.01	.05	.31	**.001**
Age	−.69	0.23	.32	.50	.79	**.003**
Adverse Childhood Experiences	−1.88	0.70	.04	.15	.59	**.007**
Parent Relationship Quality	−.57	0.38	.27	.56	1.19	.134
Peer Relationship Quality	.66	0.36	.95	1.93	3.92	.067
ACE X Parent Relationship Quality	−.31	0.51	.27	.73	2.00	.544
ACE X Peer Relationship Quality	.29	0.63	.39	1.33	4.57	.647
Subthresshold versus Full BPD						
Sex	−1.72	0.80	.04	.18	.86	**.032**
Age	−0.35	0.16	.52	.71	.96	**.027**
Adverse Childhood Experiences	−1.42	0.50	.09	.24	.64	**.004**
Parent Relationship Quality	−.44	0.28	.37	.65	1.12	.122
Peer Relationship Quality	−.45	0.24	.40	.64	1.02	.059
ACE X Parent Relationship Quality	−.74	0.34	.25	.48	.93	**.029**
ACE X Peer Relationship Quality	.26	0.39	.61	1.30	2.78	.501

Note: R^2^ = .20 (Cox & Snell), .23 (Nagelkerke). Model χ^2^ (14) = 33.27, *p* = .003. **Bold** values are significant *p* < .05

**Fig. 1:**
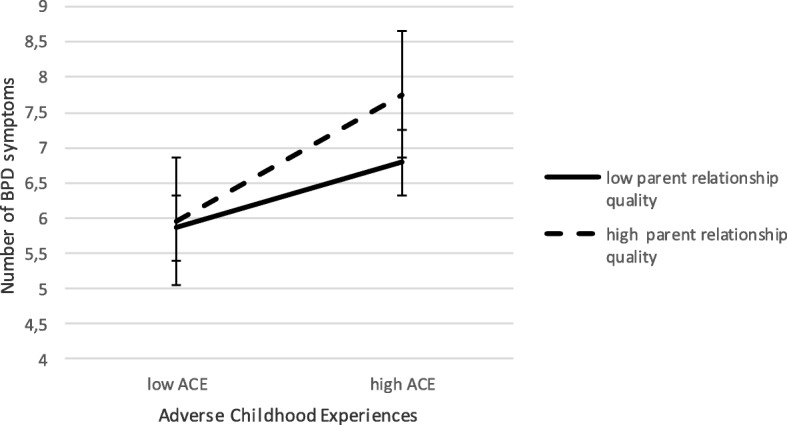
Interaction between Adverse Childhood Experiences and Parent Relationship Quality in the prediction of the number of BPD criteria

The difference between full BPD and subthreshold BPD was predicted by age (*b* = −.35, Wald *χ*^*2*^ (1) = 4.78, *p* = .027), sex (*b* = − 1.72, Wald *χ*^*2*^ (1) = 4.60, *p* = .032.), adverse childhood experiences (*b* = − 1.42, Wald *χ*^*2*^ (1) = 8.13, *p* = .004) and the interaction between adverse childhood experiences and quality of parent relations (*b* = −.74, Wald *χ*^*2*^ (1) = 4.77, *p* = .029), whereas the differences between full BPD and no BPD were predicted only by sex (*b* = − 2.95, Wald *χ*^*2*^ (1) = 10.58, *p* = .001), age (*b* = −.69, Wald *χ*^*2*^ (1) = 8.93, *p* = .003) and adverse childhood experiences (*b* = − 1.88, Wald *χ*^*2*^ (1) = 0.70, *p* = .007) and not by the interaction between adverse childhood experiences and quality of parent relations. This suggests that parent relations are of particular importance in the link between childhood adversity and BPD only for those adolescents with full BPD (i.e., ≥ 5 symptoms). A graphic presentation of the interactions between adverse childhood experiences and parent relationship quality in the prediction of the number of BPD criteria (Fig. 1) again shows that it is particularly the combination of high adversity and *good* parent relations which is related to meeting full criteria for BPD compared to subthreshold BPD.

In sum, results suggest that more adverse childhood experiences are related to more BPD. These effects hold both for the logistic regression analysis differentiating between individuals with subsyndromal and full BPD, and for the dimensional approach in which the BPD score reflects the number of BPD symptoms a participant reports. The quality of current relations with parents and peers, and the interaction between adversity and the quality of relations with peers were not related to BPD, neither in the logistic nor in the hierarchical regression analysis. However, the quality of current relations with parents moderated the link between adverse childhood experiences and BPD. This effect did not hold in the hierarchical regression analyses, but in follow-up analyses proved to be present only at full BPD compared to subthreshold BPD, showing that the combined effect of adversity and parent relationship quality is particularly relevant in those adolescents with full BPD.

## Discussion

The purpose of this study was to evaluate, in adolescents with nonsuicidal self-injury (NSSI)-disorder, whether (a) adverse childhood experiences were related to BPD, (b) current quality of social relationships with both parents and peers was related to BPD, and (c) a possible relation between the adverse childhood experiences and BPD was moderated (either buffered or aggravated) by current social relationship quality.

Overall, the results provide support for our first hypothesis concerning the relation of adverse childhood experiences and BPD: Adolescents with NSSI-disorder who reported more adverse childhood experiences showed significantly more BPD criteria and more often met full criteria for BPD. Our findings were consistent with earlier evidence that adverse childhood experiences were associated with key features of BPD [18–20]. In addition, the findings extend previous research by providing evidence for a link between adverse childhood experiences and BPD in adolescents with NSSI-disorder. Previous findings [17] showed that NSSI per se was linked to childhood adversities irrespectively of the presence of BPD*.* Our findings now demonstrated that adverse childhood adversities in general differentiated BPD from NSSI in adolescents.

The results do not provide support for our second hypothesis concerning associations between current relationships quality and BPD: no significant relations were found between current parental relationships quality and quality of peer support and BPD. This is a somewhat remarkable finding that seems in contrast to the literature [5]. The absence of significant associations between BPD and current quality of relations can be interpreted in different ways. First, it might be related to the dominance of specific diagnostic criteria at certain stages of the development [36]. More specifically, previous evidence showed that *adults* with BPD frequently report unstable relationships [36], while BPD in *adolescents* is predominantly characterized by impulsive and self-damaging symptoms, such as recurrent self harm and suicidal behaviour [37]. However, in our sample of adolescents with NSSI disorder, unstable relations as specified criterion in DSM-5 was reported by 82.8% of the group that met criteria for full BPD (versus 26.0% in the no BPD group). Therefore, an alternative explanation could be that quality of parental relations and quality of peer support do not really objectify the criterion ‘unstable relations’.

Second, the lack of link between current parental relationship quality and peer support and BPD could be interpreted as attachment figures having a greater role in socialization of emotional regulation during the first years of life compared to later developmental periods [38]. This is in line with object relations theory, which describes the internalization of early interpersonal experiences forming the building blocks for later reflective and therefore relational functioning [39], which does not imply that for example peer relationships will be less important. Third, the instability in relationships of individuals with BPD is characterized by fluctuations between extremes of idealization and devaluation [2], which could mean that especially adolescents with BPD might not accurately self-report, and in fact, might report an idealized interpretation of their current peer relationships. Fourth, in adolescents with NSSI-disorder, current quality of social relations could be impacted by impairments in social functioning, making it difficult to pick up unique links between BPD and quality of current relationships. NSSI is associated with severe impairments in relational functioning, such as interpersonal conflict [44], lack of social support [45] and in adolescents to deficits in social problem solving and communication [45].

Considering the third hypothesis, the moderating role of current social relations in the link between adverse childhood experiences and BPD, the results show a more differentiated picture. Parent relationship quality, but not peer relationship quality moderated the link between adverse childhood experiences and full vs subthreshold BPD, with particularly the combination of high adversity and good parent relationships being related to BPD. The findings show roughly comparable results from the dimensional and categorical analyses. The categorical analyses provide additional information as inverse relations between quality of early adverse experiences and parental relationships predicting BPD. Multinominal analyses have shown that this mainly differentiated full BPD and subthreshold BPD, and not BPD versus no BPD. This could imply that the moderation effect of the quality of parental relations is not linear, but only applies to the individuals on the very severe end of the borderline spectrum. However, further research may identify additional factors characterizing the person-environment interactions in adolescents at this severe end of the borderline spectrum.

Although we should be tentative in our interpretation of these findings, this matches the findings that specific childhood adversities mostly take place within a complex context and occur interrelatedly rather than independently [40]. For example, patients with BPD were found to be more likely than axis II controls to report different kinds of abuse by their caretakers and to report having caretakers deny the validity of their thoughts and feelings, fail to provide them with needed protection, neglect their physical care, withdraw from them emotionally, and treat them inconsistently [19]. In line with this, our findings showed that adverse childhood experiences were associated with less quality of parent relations. This implies that adversities might be interpreted as ‘the tip of the iceberg’ [40] indicating a complex context of more pervasive difficulties and other childhood adversities in ongoing family interactions [41, 42]. However, we found that it is particularly the combination of high adversity and *good* parent relations that was related to BPD. This seems counterintuitive and could be interpreted in different ways. First, in line with the strong negative association between quality of parental relations and adverse childhood experiences, there seemed to be only few participants who were either low on childhood adversity and low on parent relationship quality or high on childhood adversity and high on parent relationship quality. As such, these findings should be interpreted with caution. Second, the moderation effect could be interpreted in light of the difficulties in the psychosocial development of adolescents with BPD. Specifically adolescents with BPD seem to be more dependent on their parents, even when these relationships are more conflicted [43]. Therefore, particularly adolescents with BPD might rate the quality of these relations as more positive than they really are. Especially in case of adverse child experiences, adolescents at risk for BPD might develop less autonomy and stay in a more dependent relationship with their parents.

## Conclusion

Our results suggest that childhood adverse experiences have a profound role in differentiating BPD in adolescents from those with NSSI disorder, which may be stronger than current relations. Most likely, this can be explained by early life being the central phase when object relationships are formed [39] and early childhood adverse experiences are likely to set the individual on a maladaptive pathway in which they are then exposed to cumulative risks. Additionally, findings suggest that more adverse childhood experiences are related to lower quality of current relations with parents. Current relationship quality, however, was not directly related to BPD. However, when looking at the link with BPD, *higher* rather than low quality of parental relations seems to be associated with a more negative effect of adverse childhood experiences, instead of the hypothesized buffering effect. These conclusions highlight the need for extending advancements in the developmental trajectories of BPD.

There are three important limitations to this study. A first limitation is the cross-sectional design of the current study, and the related fact that childhood adversity was measured based on retrospective self-report. This can specifically be a problem because the questionnaire we used to measure adverse childhood experiences, focuses on the period prior to age 17. In a sample of adolescents of 12–17 of age, it is difficult to differentiate whether this questionnaire really focuses on *early* childhood experiences or whether the adversities actually overlap with the current relational disturbances. Especially the role of childhood adversities would be important to study in long term follow-up to further investigate how such adversities contribute to long-term outcome within a developmental pathway. To further a better understanding of the way current relationships could explain links between adverse childhood experiences and BPD, mediation analyses testing whether childhood experiences may increase risk of BPD through their impact on quality of current relationships could be used, preferably in longitudinal data. The second limitation is the self-report on the quality of relationships, which could be biased by the unstable nature of relationships and fluctuations between idealization and devaluation of their current peer relationships, which are not captured in retrospective self-report on the last week. Multi-informant report on quality of relations, for example also based on parent-report, self-report using ecological momentary assessment and including important relational aspects such as bullying might contribute to a more valid assessment of the quality of current relationships. The third limitation is that the multinomial regression makes use of small groups, which means we should be tentative in the interpretation of our results. Larger samples will be necessary to replicate our findings and study their robustness.

Despite these limitations, there are several strengths from the findings of the present study. A unique and strong point is the reliance on a consecutive clinical sample of adolescents with NSSI disorder, which allows the findings to be generalized to adolescents being at high-risk for BPD. Furthermore, the thorough assessment of the BPD criteria using semi-structured clinical interview, enabled assessing BPD both dimensionally and categorically, and both as full BPD and subthreshold BPD.

From a clinical perspective, the findings underscore the importance of improving our efforts to prevent childhood adversities, such as abuse and neglect. In addition, it confirms the need for attending to childhood adversities earlier within the developmental course by special attention to early warning signs that may arise from childhood adversities and treatment for the negative outcome of early adversities, such as childhood trauma.
